# Cancer evolution and multi-omic profile of relapsed colorectal liver metastases after treatment

**DOI:** 10.1186/s13073-026-01614-0

**Published:** 2026-02-24

**Authors:** Laura Tomás, Nathanael Raschzok, Eric Blanc, María Gallardo-Gómez, Andrea Menne, Kathy Astrahantseff, Loretta De Chiara, Dominik Geisel, Johann Pratschke, Dominik P. Modest, Angelika Eggert, Dieter Beule, David Posada, Christine Sers, Soulafa Mamlouk

**Affiliations:** 1 CINBIO, Universidade de Vigo, Vigo, 36310 Spain; 2 Galicia Sur Health Research Institute (IIS Galicia Sur), SERGAS-UVIGO, Vigo, Spain; 3 Department of Surgery, Campus Charité Mitte | Campus Virchow-Klinikum, Charité – Universitätsmedizin Berlin, corporate member of Freie Universität Berlin and Humboldt-Universität zu Berlin, Berlin, Germany; 4 BIH Biomedical Innovation Academy, Clinician Scientist Program, Berlin Institute of Health at Charité – Universitätsmedizin Berlin, Berlin, Germany; 5 Core Unit Bioinformatics (CUBI), Berlin Institute of Health at Charité – Universitätsmedizin Berlin, Berlin, Germany; 6 Translational Oncology Group, Galicia Sur Health Research Institute (IIS Galicia Sur), SERGAS-UVIGO, Vigo, Spain; 7 Department of Biochemistry, Genetics and Immunology, Universidad de Vigo, Vigo, Spain; 8 Department of Pediatric Surgery, Charité – Universitätsmedizin Berlin, corporate member of Freie Universität Berlin and Humboldt-Universität zu Berlin, Campus Virchow-Klinikum, Augustenburger Platz 1, Berlin, 13353 Germany; 9 German Cancer Consortium (DKTK), partner site Berlin, a partnership between DKFZ and Charité-Universitätsmedizin Berlin, Berlin, Germany; 10 Department of Pediatric Oncology and Hematology, Charité – Universitätsmedizin Berlin, corporate member of Freie Universität Berlin and Humboldt-Universität zu Berlin, Berlin, Germany; 11 Department of Radiology, Charité – Universitätsmedizin Berlin, corporate member of Freie Universität Berlin and Humboldt-Universität zu Berlin, Berlin, Germany; 12 Department of Hematology, Oncology, and Cancer Immunology (CCM/CVK), Charité – Universitätsmedizin Berlin, corporate member of Freie Universität Berlin and Humboldt-Universität zu Berlin, Berlin, Germany; 13 University Hospital Essen, Essen, 45147 Germany; 14 Institute of Pathology, Charité – Universitätsmedizin Berlin, corporate member of Freie Universität Berlin and Humboldt-Universität zu Berlin, Berlin, Germany

**Keywords:** Subclone, Resistance, Immune-cell infiltration, Neoantigens

## Abstract

**Background:**

Recurrence following resection of colorectal cancer liver metastases remains a major obstacle to prolonged patient survival, often resulting in treatment-refractory disease with limited understanding of the underlying evolutionary drivers. To investigate these mechanisms, we performed an in-depth, patient-specific study of genomic and microenvironmental alterations in relapsed colorectal cancer liver metastases from nine individuals.

**Methods:**

Clonal deconvolution and phylogenetic analysis was conducted on DNA sequencing data from multiregion liver metastasis and relapse samples from the same patient. Archived primary tumor specimens were included to trace the clonal lineages responsible for relapse. In parallel, transcriptomic data from the liver metastasis and relapse samples were analyzed to characterize tumor-infiltrating immune cell populations.

**Results:**

Phylogenetic analyses of relapsed metastases revealed two distinct patterns: (1) relapses that retain a clone already present in ancestral liver metastasis, and (2) relapses with no evident clonal link to the original metastasis. Relapses in the first group carried a chemotherapy-associated mutational signature, which then appeared across all relapse samples. In one patient, the relapsing clone had already diversified within the primary tumor. Tumor microenvironment analyses exposed heterogeneous responses followwing relapse, ranging from decreased to markedly increased infiltration by multiple immune cell types, accompanied by shifts in consensus molecular subtypes and altered neoantigen profiles.

**Conclusions:**

Our study reveals patient-specific evolutionary trajectories underlying relapse in colorectal cancer, highlighting diverse routes to recurrence, including chemotherapy-driven clonal expansion and early divergence from the primary tumor. Our findings reveal a landscape of patient-specific evolutionary trajectories in relapsed colorectal cancer, underscoring the potential value of personalized approaches for understanding and monitoring recurrent disease.

**Supplementary Information:**

The online version contains supplementary material available at 10.1186/s13073-026-01614-0.

## Background

Colorectal cancer (CRC) is the second leading cause of cancer mortality worldwide [1], and more than half of patients diagnosed with CRC will develop liver metastases. International consensus guidelines suggest systemic therapies chosen on basis of tumor-specific information (molecular subgroups and primary tumor location) and clinical presentation of the patients [2]. Colorectal liver metastases (CRLM) are treated directly or following systemic therapy by local treatment (mainly surgery), which might be combined or partly replaced by local ablative techniques [3–5]. The role of adjuvant/additive therapy after local treatment of CRLM remains unclear [2]. Despite some improvements in response to treatment over the past years[6], 50–75% of patients with CRLM relapse after treatment within the first two years of diagnosis [7–11]. The metastatic relapse is typically managed with the same therapeutic approaches used for the initial metastasis [12], often with limited improvement in patient outcomes [13–15]. During repeated treatment, patients can develop multidrug resistance to systemic therapy, providing a compelling rationale to discontinue treatment. While immunotherapy, in the form of checkpoint inhibitors, has improved the treatment of microsatellite instable (MSI) or mismatch repair-deficient CRCs [16], the majority of CRCs are microsatellite stable (MSS) and are not treated with immunotherapy [17].

Although generally considered a cold tumor with limited responsiveness to immunotherapy, tumor-infiltrating immune cells are abundant in MSS CRC and correlate with a lower risk of recurrence at five years [18] as well as improved clinical outcomes [19]. Studies on infiltrating leukocytes [18, 20] and the consensus molecular subtypes (CMS) [21] also demonstrate that the tumor microenvironment can be altered following treatment [22–25]. However, information remains limited on later stages of relapsed CRC, although treatment with cytotoxic agents has been shown to induce tumor immune response in several cancer types [17, 26].

Genetic alterations play a key role in driving the initial CRC progression [27–30], but their importance in later stages of the disease remains unclear [31–34]. The limited studies conducted on relapsed CRC have shown few additional genomic changes in the relapse tumor. In one study, four matched samples (metastasis and relapse from the same patient) showed almost identical mutational profiles before and after relapse [29]. Likewise, similar mutational profiles were found in multiple CRC metastases from different organs, including two patients with multiple liver metastases [35]. In a study using unmatched patient samples to analyze copy number alterations before and after CRLM relapse, there were no significant differences, apart from a gain in chr18p11.32 enriched in the relapses [36]. In a study of unresectable CRLMs, microenvironmental factors were suggested to be responsible for the relapse, although *SMAD4* p.R361H/C mutations and chr20q gains were also linked to treatment resistance [37]. In our previous in-depth study of CRLM and matched relapse in one patient using single-cell DNA only four additional non-synonymous mutations were present in the relapse after three years of post-resection treatment, none of which had a known implication [38].

While several studies have suggested that CRC metastases can occur early and before microscopic detection [39, 40], evidence that removal of early adenomas can reduce disease progression [41, 42] argues against early metastatic seeding in the majority of patients. Investigations utilizing single-cell DNA sequencing have yielded mixed results as well, indicating both late dissemination with monoclonal seeding and polyclonal seeding in different patients [43–46]. How metastatic seeding evolves at late stages of relapsing disease remains unknown.

Importantly, whether relapse following multiple cycles of neo-adjuvant and/or adjuvant therapy and surgery stems from existing resistant cells in the metastases, or whether new driver alterations promote new subclones to relapse remains unclear. A better understanding of post-resection relapse tumor is crucial to refine therapeutic strategies and improve patient outcomes. Here, we reconstructed the evolutionary history of relapsing CRLM and characterized the tumor immune microenvironment using multiple, pre-treatment and post-treatment samples from nine patients. We additionally utilized archived primary tumor material from five patients to track the origin of relapsing clones.

## Methods

### Patient tissue samples and ethical approval

Frozen liver and tumor tissue samples from nine patients undergoing consecutive open liver resections for treatment of primary and relapsing CRLM from 2004 to 2013 in the Department of Surgery at the Charité—Universitätsmedizin Berlin were analyzed, according to the ethics vote EA1/187/16. Clinical histories were collected retrospectively. All cases were reviewed by an independent radiologist to confirm that the relapsing metastasis was not caused by local recurrence of the primary metastasis. Multiple samples from one tumor tissue were frozen separately in liquid N2. Samples were stored at minus 80 °C. In total, 117 samples were isolated and analyzed: 63 samples for whole exome sequencing (6 tumor samples; 3 from the initial CRLM, labeled ‘L’, and 3 from the matched relapsed CRLM labeled ‘LR’, and 1 healthy sample per patient), and 54 samples for RNA sequencing (6 tumor samples). In addition, primary tumor tissue material from *Patients 2, 5, 6, 7,* and *MSI* was isolated from Formalin-fixed paraffin-embedded (FFPE) blocks.

### DNA and RNA isolation

Frozen tissue sections were sectioned with a cryostat and placed into a cold tube. DNA and RNA from the same sample were isolated using the AllPrep DNA RNA Kit (Qiagen, Netherlands). DNA and RNA were quantified with Qubit Assays (Thermo Fisher Scientific, USA), and the quality was determined with TapeStation (Agilent Technologies, USA). For the FFPE samples, tissue was macro-dissected from the slides, and DNA was isolated using the GeneRead DNA FFPE kit (Qiagen, Netherlands). The quality and quantity of DNA were determined by TaqMan RNaseP Detection Reagents Kit (Thermo Fisher Scientific, USA).

### Sequencing of genomic material

#### Whole Exome Sequencing (WES)

Libraries were generated using the SureSelectXT Automation Reagent Kit and SureSelectXT Human All Exon v6 Capture Library (Agilent Technologies), following the manufacturer’s instructions. In brief, 200 ng of gDNA was fragmented to ~ 150 bp using a Covaris LE220 ultrasonicator (Covaris, Inc.). Subsequently, library preparation was performed on a Bravo automated liquid handler (Agilent Technologies) including end-repair, A-tailing, adaptor ligation, and amplification. The concentration of the amplified, adaptor-ligated DNA library was determined using the TapeStation (Agilent Technologies). In the subsequent steps 750 ng of amplified, adaptor-ligated DNA library was used for the hybridization reaction with the SureSelectXT All Exon v6 bait set. The DNA-library/bait hybrids were captured using streptavidin-coated magnetic beads (Dynabeads MyOne Streptavidin T1 by Thermo Fisher Scientific). Index tags were added in the course of PCR-amplification of the captured libraries. 2 × 100 bp paired-end sequencing at ~ 450X was performed on the Illumina HiSeq 4000 according to the manufacturer's protocol.

#### RNA sequencing

Sequencing libraries were prepared using the Illumina TruSeq mRNA stranded Kit following the manufacturer's instructions. Briefly, mRNA was purified from 500 ng of total RNA using oligo(dT) beads. Then poly(A) + RNA was fragmented to 150 bp and converted to cDNA. The cDNA fragments were then end-repaired, adenylated on the 3′ end, adapter-ligated, and amplified with 15 cycles of PCR. Quality control of the final libraries was performed using Qubit (Invitrogen) and Tapestation (Agilent Technologies). 2 × 100 bp paired-end sequencing was performed on the Illumina HiSeq 4000 according to the manufacturer's protocol. This produced between 96,296,575 and 197,551,406 read pairs per sample (median 131,730,506).

### DNA analysis

#### NGS DNA data preprocessing

The quality control of the sequencing reads was performed using FASTQC [47] (version 0.11.7). The Illumina sequencing adapters were removed using Cutadapt [48] (version 1.18) and the reads were mapped to the human reference genome hs37d5 using BWA mem [49] (version 0.7.17). Following the Genome Analysis Toolkit (GATK) Best Practices [50], base quality scores were recalibrated using GATK (version 4.1.2.0) BaseRecalibrator and ApplyBQSR. Contamination from other individuals was estimated using GATK CalculateContamination.

#### SNV and indel calling

Single-nucleotide variants, insertions, and deletions (SNVs and indels, from hereon collectively termed mutations) were called using GATK Mutect2 [51] (version 4.1.3.0) in multi-sample mode (joint calling), performing one run for each patient. The joint analysis included the healthy sample as a control and all the tumor samples from the metastasis, the relapse, and the primary tumor when available. An in-house panel of normals, the SureSelect exome panel target, and the Gnomad database file [52] were provided to help remove possible artifacts and germline SNPs. The -f1r2 option was used to collect read pair orientation metrics and the read filter MateOnSameContigOrNoMappedMateReadFilter was disabled as recommended by the GATK authors. GATK LearnReadOrientationModel (version 4.1.3.0) was then used to get the maximum likelihood estimates of artifact prior probabilities in the orientation bias mixture model filter. The calls were filtered using GATK FilterMutectCalls, incorporating the model derived from LearnReadOrientationModel and the contamination estimates from GATK CalculateContamination. Mutations were annotated using Annovar [53] (version 2018Apr16) with the databases refGene, cosmic70, and avsnp150. A mutation was considered present in a sample if it had a variant allele frequency (VAF) ≥ 0.05 and at least one alternative read. A list of driver genes was obtained from IntOGen [54] and the non-silent mutations affecting those genes were classified as "mutations in driver genes".

#### Copy number calling

Allele-specific somatic copy number alterations (SCNAs) and tumor purity were inferred using Sequenza [55] (version 3.0.0). The standard Sequenza pipeline was followed for each tumor and its matched healthy sample. First, a genome-wide GC content file in 50-bp windows was created using sequenza-utils gc_wiggle and the reference genome. The seqz files were generated using sequenza-utils bam2seqz, providing the healthy and tumor BAM files and the GC content file as inputs. Then, sequenza-utils seqz_binning was run with a window size of 50 bp. The depth ratio was normalizd for GC content, and allele-specific segmentation was performed using sequenza.extract. The log-posterior probability for all candidate combinations of purity and ploidy was calculated with sequenza.fit. Finally, the results were exported with sequenza.results.

#### Clonal deconvolution

Clonal deconvolution was performed using PyClone-VI [56] (version 0.1.1) and LICHeE [57] (version 1.0), integrating information from primary tumor, metastasis, and relapse samples. For this, biallelic mutation calls from Mutect2 were used, considering them present in a sample if they were supported by more than five alternative reads. Mutations present in at least one liver sample and with depth ≥ 100 in all the samples were selected. Mutations found exclusively in the primary tumor samples were excluded, as FFPE-derived sequences are prone to false positives [58]. This filtering approach implies that clones private to the primary tumor won't be detected. Copy number and tumor purity values from Sequenza were added, and those variants for which allele-specific copy number states were not available were removed. PyClone-VI was run using 100 restarts, a maximum of 15 mutation clusters, and a beta-binomial density model. Mutation clusters with more than 5 mutations and prevalence ≥ 0.2 in at least one sample were selected, and were considered present in a sample if their prevalence was at least 0.1. From those mutation clusters, trees were built using LICHeE with options -clustersFile, -e 0.25, and -maxClusterDist 0.1. The outputs were parsed with an in-house script and only the top-score tree for each patient was retained. The most ancestral cluster inferred in all the samples but not in all the metastasis samples was identified, and the first clone harboring it was termed the "relapsing clone".

#### Sample tree reconstruction

To cross-validate the clonal deconvolution results, sample trees were reconstructed using Treeomics [59] (version 1.9.2). Mutations with VAF ≥ 0.05 in at least one sample and depth ≥ 100 in all samples were selected, excluding those detected only in the primary tumor sample or located in regions with copy number loss for any samples. Treeomics was run with options -e 0.01, -af 0.05, and –wes_filtering, using the total depth and alternative allele counts of the selected mutations together with the tumor purity of each sample.

#### Copy-number based sample tree reconstruction

Copy number sample trees were reconstructed using MEDICC2 [60] (version 1.1.2). Primary tumor samples were excluded from this anaysis due to the unreliability of the copy number profiles derived from FFPE sequencing [61]. Sequenza copy number calls for the liver samples were first jointly resegmented using Refphase [62] (version 0.3.2). Following the author's recommendations, heterozygous SNPs were extracted from the Sequenza seqz output using the load_snps_seqz function, BAF and logr values were adjusted using center_baf and fit_logr_to_ascat, and Refphase was run using the method "ascat" to re-estimate the copy number when required. Then, copy number trees were built using MEDICC2 with a minimum segment length of 1 Mb and the event reconstruction option enabled. If the samples containing the relapsing clone from LICHeE formed a monophyletic group in the MEDICC2 tree, the ancestral branch leading to that clade was defined as compatible with the relapsing clone.

#### Mutational signatures

Mutational signatures in the liver samples were inferred using sigProfiler [63] (version 1.1.16). First, multi-sample VCFs were split into single-sample VCFs using bcftools [64] (version 1.12). VCFs were filtered to keep only the mutations present in each sample (VAF ≥ 0.05 and more than one alternative read). Then, sigProfilerExtractor was run jointly for all the MSS patients and independently for the MSI patient. For this, COSMIC single-base substitution (SBS) signatures version 3.3 were used, with the maximum number of signatures set to ten, the number of replicates to 100, and the WES mode enabled.

### mRNA analysis

#### Next generation sequencing RNA data preprocessing

Transcript read counts were obtained using salmon [65] (version 1.4.0) in quantification mode, using the mapping validation option and the automatic detection of protocol strandedness. Quantification was made based on the human genome reference sequence GRCh38.d1.vd1 provided by NIH's Genomic Data Commons, which includes decoys and virus sequences. The genome was annotated with features from the GENCODE version 33 primary assembly. The contig names of 717 features on unplaced and unlocalized contigs were renamed to match the naming conventions used in GRCh38.d1.vd1.

The complete transcriptome based on the GENCODE 33 annotation was created using the rsem-prepare-reference from rsem tools [66] (version 1.2.28). Salmon indices were then prepared for a kmer length of 31 by merging those transcript sequences with the whole reference sequence used as a decoy, following salmon's authors' recommendations.

#### mRNA expression analysis

Transcript read counts computed by salmon were merged into gene read counts using the Bioconductor package tximport [67] (version 1.18.0). These values were then supplied to the Bioconductor package DESeq2 [68] (version 1.30.1) and normalized by variance-stabilisation. The normalization was done separately for each patient, except for one additional normalization which was done on the whole cohort. These normalized expression values were then used to compute single-sample gene-set enrichment scores, based on established profiles for tumor-infiltrating leukocytes [69] and the ssGSEA method implemented in the Bioconductor package GSVA [70] (version 1.40.1). Gene sets statistical significance was computed using the CERNO test [71].

#### CMS classification

The R package CMScaller [72] (version 2.0.1) was used on unnormalized gene expression counts. Here, profiles from Guinney et al. [21] were used to define CMS classes.

#### Fusion genes

Detection of gene fusions was carried out using STAR [73] (version 2.7.10a) and Arriba [74] (version 2.3.0). STAR indices were computed for genome GRCh38.d1.vd1 using the GENCODE version 33 primary assembly as feature annotation database for exon junctions. The same contig renaming was carried out as for the preliminary steps of mRNA processing. STAR parameters were set following Arriba documentation, and Arriba was run with default settings.

To reduce the rate of false positives in the downstream analysis, only candidates flagged as “high confidence” by Arriba and with more than one split read (for both mates) and one discordant read supporting the fusion were retained. Out of 908 candidates, 129 candidates remained after filtering.

#### Epitope prediction for neoantigens

pVACseq [75] pipeline was employed to predict cancer peptides within our samples, utilizing information from both DNA and RNA. First, the *HLA* alleles for each patient were obtained by mapping the mRNA and DNA sequencing reads on *HLA* locus with yara [76] (version 1.0.2) and then running the OptiType pipeline [77] (version 1.3.5). Then, neo-epitope prediction from the SNVs was conducted using pVACseq [75] (version 3.1.2, docker digest 2c74c1eaa06a) and specifying the patients' *HLA* alleles. Predictions were made with all type I tools for epitopes of length nine to 12, using the blastp database provided with the pVACseq docker image (selected refseq proteins).

For each tumor sample, the somatic VCF obtained from the DNA analysis was completed with the support for the SNVs in the corresponding RNA sample. For this, STAR was first used to map the RNA reads to the reference genome hs37d5, since the somatic variant calling was done using this genome release. Then, the number of RNA reads supporting the reference and alternative alleles at the SNV loci were obtained with bcftools [78] (version 1.17). The gene expression abundance was computed using salmon, and added to the augmented VCF as transcripts per million.

## Results

### Cohort description

To investigate the evolution of relapsing CRC, we conducted a multi-omic analysis encompassing whole-exome sequencing and RNA sequencing of a well-defined sample cohort from nine patients with CRLM. Our cohort comprised eight patients with the MSS CRC subtype, including cases from both rectal (*Patients 3*, *5*, *7*, and *8*) and colonic (*Patients 1*, *2*, *4*, and *6*) origins, and one patient with the MSI subtype, *Patient MSI*. All patients have been routinely followed by quarterly CT and/or MRI scans after the initial liver resection. The average time to relapse was 13 months (Table 1). All patients have undergone 5FU-based chemotherapy, either neoadjuvant or adjuvant and with or without the administration of a VEGF inhibitor (bevacizumab). *Patient 3* and *Patient 5* received treatment before their primary tumor was resected, specifically folinic acid, fluorouracil, and oxaliplatin (FOLFOX) with bevacizumab, or radiation, respectively (detailed clinical information is available in Additional file 1: Fig. S1 and Additional file 2: Table S1). Liver metastases and relapse were always found in separate regions of the liver (CT scans of liver segments of tumor locations can be found in Additional file 1: Fig. S2).

**Table 1:** Overview of sample cohort

Patient	Primary tumor site	Diagnosis pattern	Time between primary and CRLM OP (mo)	Time to relapse (mo)	Therapy before primary tumor resection	Therapy before CRLM resection	Therapy before relapse resection
Patient 1	Ascending colon	Metachronous	27	32	None*	5FU, Oxaliplatin	FOLFIRI
Patient 2	Ascending colon	Synchronous	0	13	None	None	Oxaliplatin, 5FU
Patient 3	Rectum	Synchronous	31	11	FOLFOX, Bevacizumab	5FU	None
Patient 4	Ascending colon	Synchronous	5	14	None	FOLFOX, Bevacizumab	None
Patient 5	Rectum	Metachronous	13	7	Radiation	5FU	FOLFOX
Patient 6	Descending colon	Metachronous	13	13	None	Irinotecan, 5FU	FOLFOX
Patient 7	Rectum	Metachronous	23	14	None	FOLFOX, Irinotecan, Bevacizumab	FOLFIRI
Patient 8	Rectum	Metachronous	1	11	None	None	Xeloda
Patient MSI	Colon transverse	Synchronous	8	16	None	FOLFOX, Avastin	FOLFOX, Avastin

*OP* Operation, *mo* Months. *only surgery was performed. *FOLFOX* Folinic acid, fluorouracil, and oxaliplatin, *5FU* 5-Flourouracil, *FOLFIRI* Folinic acid, fluorouracil, and irinotecan

**Fig. 1:**
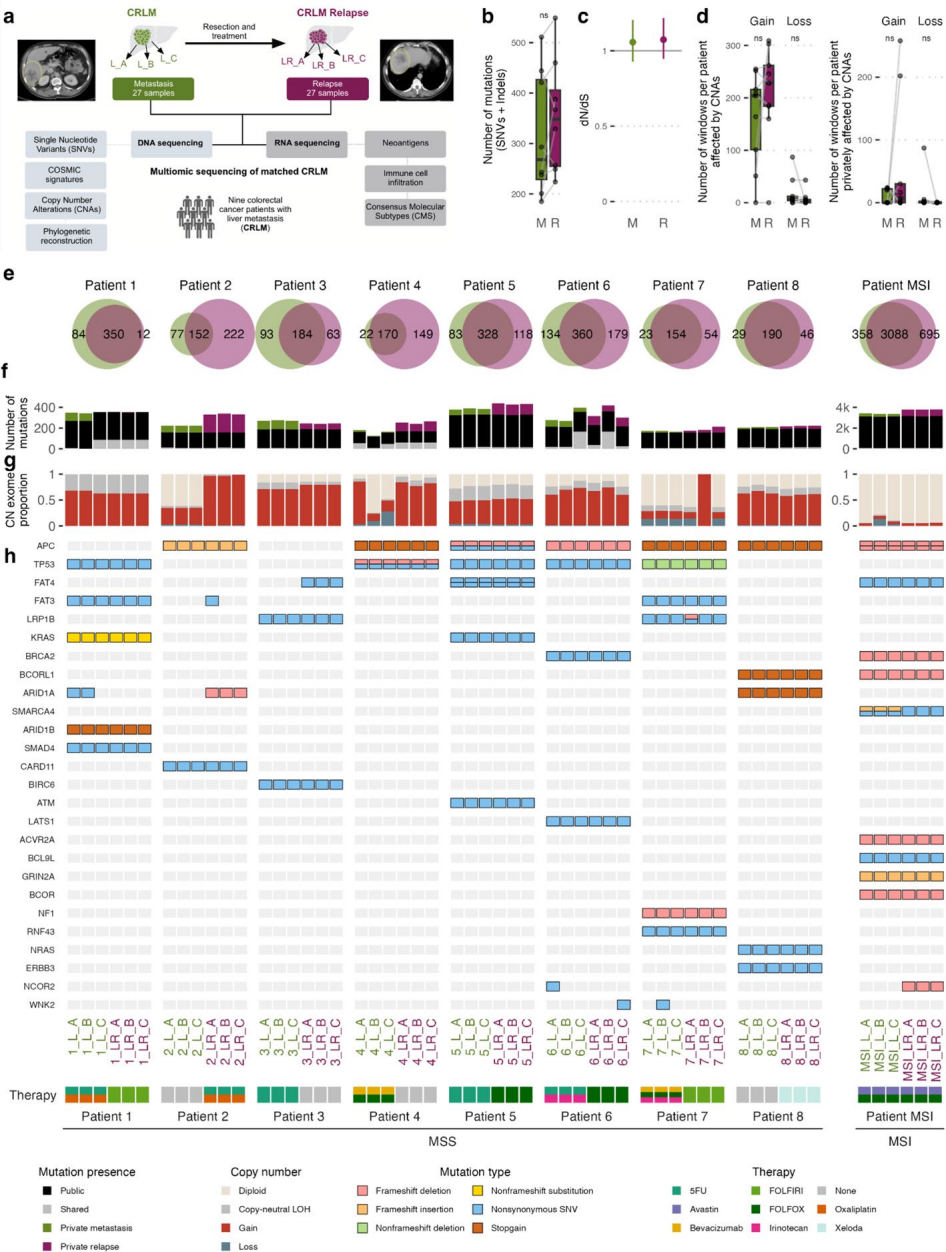
Study design and mutational landscape of relapsing CRLMs. **a** Experimental overview. Colorectal cancer liver metastases (CRLM) from nine patients, with three samples from the metastasis (L_A, L_B, and L_C, in green) and three from the relapse (LR_A, LR_B, and LR_C, in purple) were investigated using DNA whole-exome sequencing and RNA sequencing. **b** Total number of mutations in metastasis (M) and relapse (R) samples per MSS patient. Significance level for paired Wilcoxon test. **c** dN/dS (ratio of non-synonymous to synonymous mutations) for the mutations found in the metastasis (M) and the relapse (R) samples of the MSS patients. **d** On the left, number of 10-Mb genomic windows per MSS patient affected by copy number alterations (SCNAs) in the metastasis (M) and the relapse (R) samples per patient. On the right, number of 10-Mb genomic windows per MSS patient affected by SCNAs only in the metastasis (M) or only in the relapse (R) samples. In the boxplots the central line indicates the median, while the box limits correspond to the Q1 and Q3 quartiles; upper and lower whiskers extend from Q3 to Q3 + 1.5 × (Q3-Q1) and from Q1 to Q1—1.5 × (Q3-Q1), respectively. Significance levels for paired Wilcoxon test. **e** Venn diagram depicting the total number of mutations per patient and proportion of overlap between metastasis and relapse. Only mutations covered by more than 20 reads across all samples from a patient are shown. **f** Number of mutations per sample. Colors indicate whether the mutations are public (present in all the samples from the patient), private to the metastasis (present only in one or multiple samples from the metastasis), private to the relapse (present only in one or multiple samples from the relapse), or shared (present in both metastasis and relapse samples, but not public). **g** Per-sample overview of the proportion of the exome copy number altered.** h** Non-silent mutations in colorectal carcinoma (COREAD) compendium driver genes. The bottom panel indicates the treatment administered before each time point

For each patient, we collected three CRLM samples before liver relapse (denoted L_A, L_B, and L_C; Fig. 1a); three samples after liver relapse (LR_A, LR_B, and LR_C), and samples of normal surrounding tissue. In total, we investigated 117 samples. For *Patients 2, 5, 6, 7*, and *MSI*, we also obtained FFPE blocks from patients’ primary tumor to trace back the origin of the relapse. The primary tumor samples are denoted with P_A.

### The genomic landscape of relapsing CRLM

To identify genetic differences between metastasis and relapse tumors in CRC, we first compared DNA alterations in CRLM samples and relapse samples (Additional file 3: Table S2). There was no significant change in the numbers of single nucleotide variations, insertions, and deletions (SNVs and indels, from hereon collectively termed mutations) between the metastasis and relapse samples (paired Wilcoxon test *p* = 0.18, mean ± s.d. = 317 ± 124 in metastasis and 353 ± 112 in relapse; Fig. 1b). In addition, we found no significant changes in selective pressure by ratio of non-synonymous versus synonymous mutations (dN/dS [79]) among metastasis and relapse samples (Fig. 1c and Additional file 1: Fig. S3a). Even so, *Patient 2* and *Patient 4* had many mutations exclusively in the relapse samples (222 and 149 mutations, respectively, in positions covered at least 20X across all samples), and *Patient 3* had 93 mutations present solely in the metastasis samples (Fig. 1e). However, mutation burden correlated with tumor cell content (Pearson R = 0.29, *p* = 0.043 for the MSS patients), and for *Patient 4* the tumor cell content was noticeably lower in the metastasis samples than in the relapse (Additional file 1: Fig. S3b). *Patient 1* had only 12 mutations exclusively in the relapse, but the relapse samples shared 85 mutations with the metastasis sample 1_L_C (Fig. 1f). *Patient* 6 had 131 mutations shared between the metastasis sample 6_L_C and the relapse sample 6_LR_B. Similarly, the burden of SCNAs did not change significantly after relapse (Wilcoxon test p = 0.53 for gains and *p* = 0.21 for losses; Fig. 1d). *Patient 2*, *Patient 4*, and *Patient 7* showed remarkable differences in copy number burden among samples (Fig. 1g). As expected, *Patient MSI* showed more mutations (4,227) and a smaller proportion of the genome affected by copy number changes than patients with MSS CRC (last column in Fig. 1f, g).

The majority of non-silent mutations in *colorectal adenocarcinoma compendium driver genes* (COREAD) [54] were detected in both the matched metastasis and relapse samples (Fig. 1h). However, some mutations in COREAD genes were present in only a subset samples. For example, two metastasis samples of *Patient 1* (1_L_A and 1_L_B) had a mutation in *ARID1A* A2181P, while samples from *Patient 2, Patient 3*, and *Patient MSI* presented mutations shared exclusively among the three relapse samples (*ARID1A* A905fs for *Patient* 2, *FAT4* Q4873H for *Patien*t *3*, and *NCOR2* P957fs for *Patient MSI*). There were also mutations detected in a single sample from a patient in *FAT3* (sample LR_A from *Patient 2*), *LRP1B* (sample LR_A from *Patient 7*), *NCOR2* (sample L_A from *Patient 6*), and *WNK2* (sample LR_C from *Patient 6* and sample L_B in *Patient 7*). When exploring all non-synonymous mutations, including non-COREAD genes, only 12 genes appeared mutated in more than two patients with MSS CRC (Additional file 1: Fig. S3c). Genes *ADGB*, *MUC5B*, and *MUC4* were mutated in four patients with MSS CRC.

### CRLM with relapsing clones present in the first metastases contain a chemotherapy-induced DNA signature

**Fig. 2:**
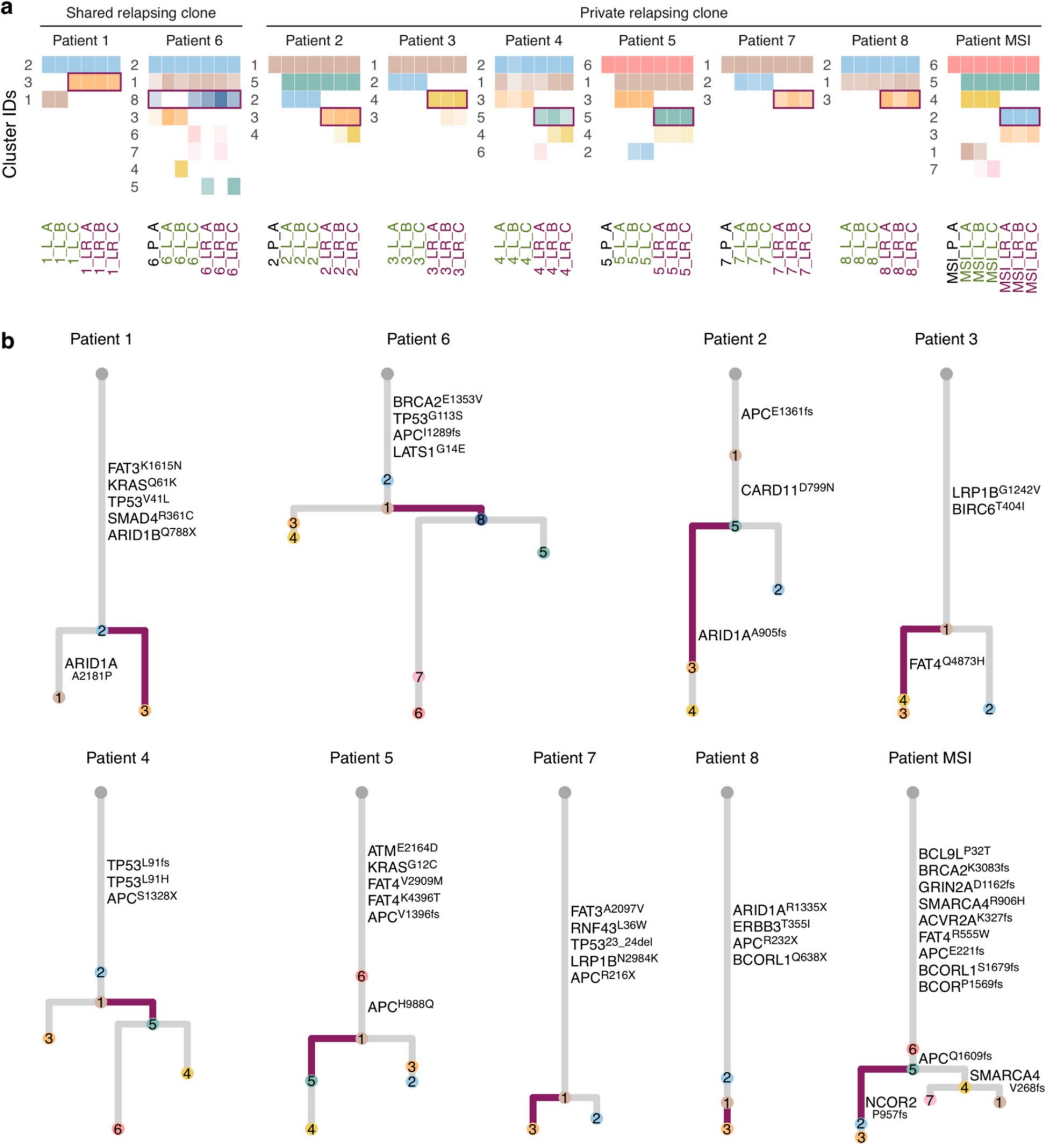
Evolution of relapsing CRLM. **a** Mutation clusters inferred with clonal deconvolution. This analysis includes FFPE primary tumor samples when available, but only mutations detected in at least one liver sample were retained. Color depth depicts the frequency of the cluster in the sample. Clusters defining the relapsing clone are outlined in purple. Patients are grouped into those in which the relapsing clone was already observed in the metastasis samples (shared relapsing clone, *Patient 1* and *Patient 6*) and patients with no evidence of the relapsing clone in the metastasis samples (private relapsing clone, *Patients 2*, *3*, *4*, *5*, *7*, *8*, and *MSI*). **b** Phylogenetic trees illustrating the evolutionary relationships among all clones identified in each tumor. Colors match cluster IDs in (**a**). Branches leading to the relapsing clones are depicted in purple

We reconstructed the evolutionary history of each tumor and found that all relapses originated from a single clone. We identified mutation clusters (sets of mutations at the same frequency) present across all relapse samples but absent from some metastasis samples and designated the most ancestral clone with these clusters as the "relapsing clone" (Fig. 2a, Additional file 1: Fig. S4a, and Additional file 4: Table S3). We identified two distinct patterns. In two patients (*Patient 1* and *Patient 6*), the relapsing clone was present in one of the three metastasis samples, a pattern we will refer to as "shared relapsing clone". In the remaining seven patients, the relapsing clone was detected only in the relapse samples, a pattern we termed "private relapsing clone". For patients with primary tumor samples available, we investigated the possible dissemination trajectory. In *Patient 6*, we found evidence of two low-prevalence clones in the primary: the relapsing clone (*Clone 8*), and another clone also present in two metastasis samples but not detected in the relapse (*Clone 3*). For the other patients, the relapsing clone was not detected in the primary tumor. In *Patient 2*, *Patient 5*, and *Patient MSI*, there was a clone shared among all the liver samples that did not appear in the primary tumor (*Clone 5*, *Clone 1*, and *Clone 5*, respectively), suggesting a common origin of metastasis and relapse. In *Patient 7*, the metastasis and relapse clones directly diverged from the primary tumor, a pattern compatible with an independent seeding. To cross-validate the evolutionary analysis, we built sample trees using Treeomics (Additional file 1: Fig. S5). Although sample trees can not capture complex evolutionary trajectories like the diverging primary tumor clones in *Patient 6*, the genetic similarity among samples was highly compatible with the relapsing clone patterns observed with clonal deconvolution.

Most mutations in COREAD driver genes were clonal (present in all the tumor cells; Fig. 2b). For the patients with MSS CRC, these included mutations in *APC* (p.I1289fs in *Patient 6*, p.E1361fs in *Patient 2*, p.S1328X in *Patient 4*, p.H988Q and p.V1396fs in *Patient 5*, p.R216X in *Patient 7* and p.R232X in *Patient 8*), *TP53* (p.V41L in *Patient 1*, p.G113S in *Patient 6*, p.L91fs and p.L91H in *Patient 4* and p.23_24del in *Patient 7*), *FAT4* (p.V2909M and p.K4396T in *Patient 5*), *FAT3* (p. K1615N in *Patient 1* and p.A2097V in *Patient 7*), *LRP1B* (p. G1242V in *Patient 3* and p.N2984K in *Patient 7*), *KRAS* (p.Q61K in *Patient 1* and G12C in *Patient 5*), *BRCA2* (p.E1353V in *Patient 6*), *BCORL1* (p.Q638X in *Patient 8*), *ARID1A* (p.R1335X in *Patient 8*), ARID1B (Q788X in *Patient 1*), *SMAD4* (p.R361C in *Patient 1*), *LATS1* (p.G14E in *Patient 6*), *CARD11* (p.D799N in *Patient 2*), *BIRC6* (p.T404I in *Patient 3*), *ATM* (p.E2164D in *Patient 5*), *RNF43* (p.L36W in *Patient 7*), and *ERBB3* (p.T355I in *Patient 8*). In addition, we identified relevant subclonal mutations. When looking at the mutations defining the relapsing clones, we found non-silent mutations in COREAD driver genes for *Patient* 2 (*ARID1A* p.A905fs), *Patient 3* (*FAT4* p.Q4873H), and *Patient MSI* (*NCOR2* p.P957fs). Nevertheless, most relapsing clones did not harbor any COREAD driver mutations. After expanding our search to non-COREAD genes, we found that the shared relapsing clone in *Patient 1* contained a non-synonymous SNV in *DLGAP2* (p.G336R), previously reported in a CRC patient from COSMIC (COSMIC IDs COSM1552255 and COSM1552254; Additional file 1: Fig. S4b and Additional file 3: Table S2). The private relapsing clone in *Patient 2* harbored a stop gain mutation in *LRRC4C* p.R156, already reported in COSMIC for CRC and liver cancer (COSM187765). When looking at the samples without the relapsing clone, we observed a mutation in the COREAD genes *ARID1A* (p.A2181P) for *Patient 1* and in SMARCA4 (p.V268fs) for *Patient MSI*.

**Fig. 3:**
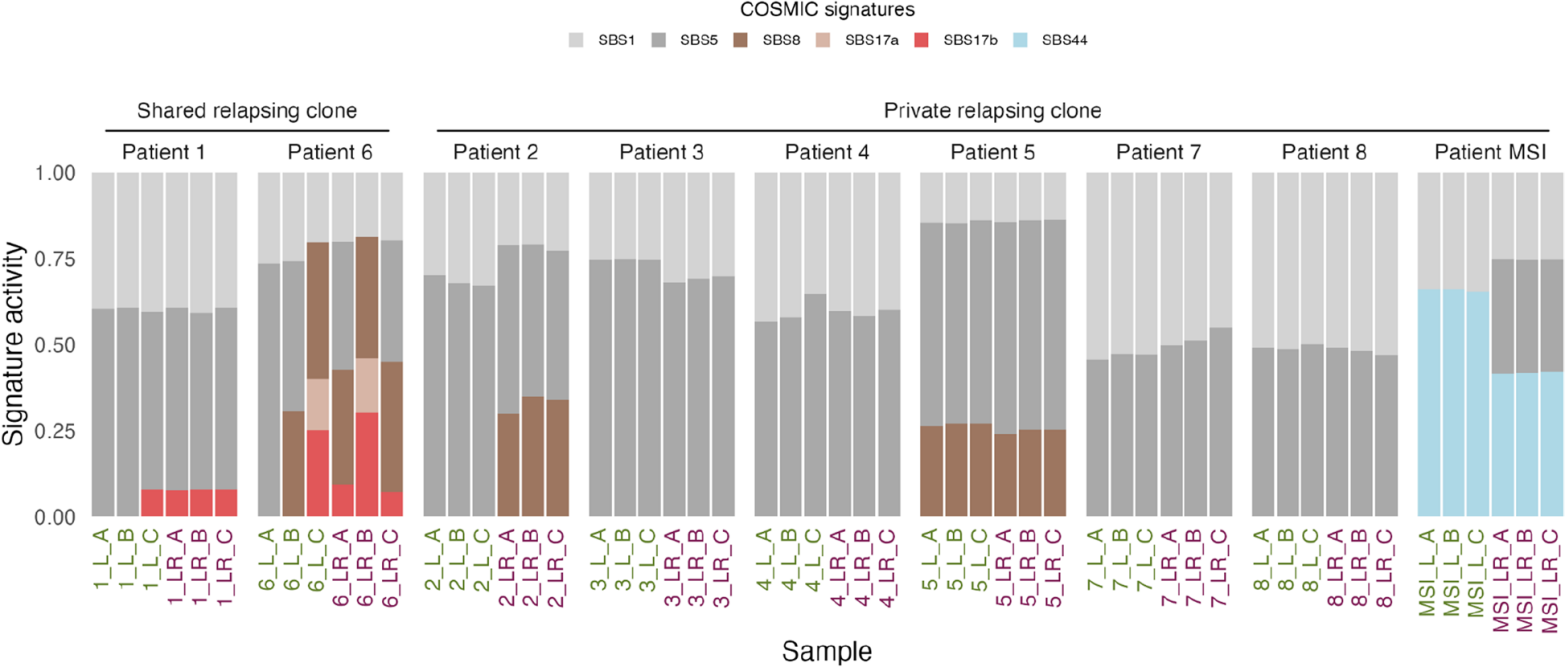
COSMIC DNA SBS signatures before and after relapse. Mutational signatures detected in metastasis (green labels) and relapse (purple labels) liver samples. DNA signature SBS1: deamination of 5-methylcytosine; SBS5 and SBS8: unknown etiology; SBS17a: damage by ROS; SBS17b: damage by ROS and 5FU chemotherapy; SBS44: defective DNA mismatch repair

All samples shared the clock-like mutational signature SBS1 (Fig. 3). SBS5, another clock-like signature, appeared in all samples except the metastasis samples of *Patient MSI*, 6_L_C, and 6_LR_B*. Patients 2*, *5*, and *6* showed the SBS8 signature (unknown etiology). In addition, two samples in *Patient 6* presented SBS17a (damage by ROS). Intriguingly, our analysis revealed that the metastasis samples carrying the shared relapsing clones in *Patient 1* and *Patient 6* (1_L_C and 6_L_C) shared the SBS17b signature with their relapse samples. This signature has been linked to 5FU chemotherapy and ROS damage. Both these patients received 5FU treatment as neoadjuvant and adjuvant treatment (Table 1). *Patient 6* was reported to initially respond to neoadjuvant 5FU and was treated with ablation, but progressed again and underwent a resection of the first metastasis.

### DNA copy number changes in relapsing characteristics

**Fig. 4:**
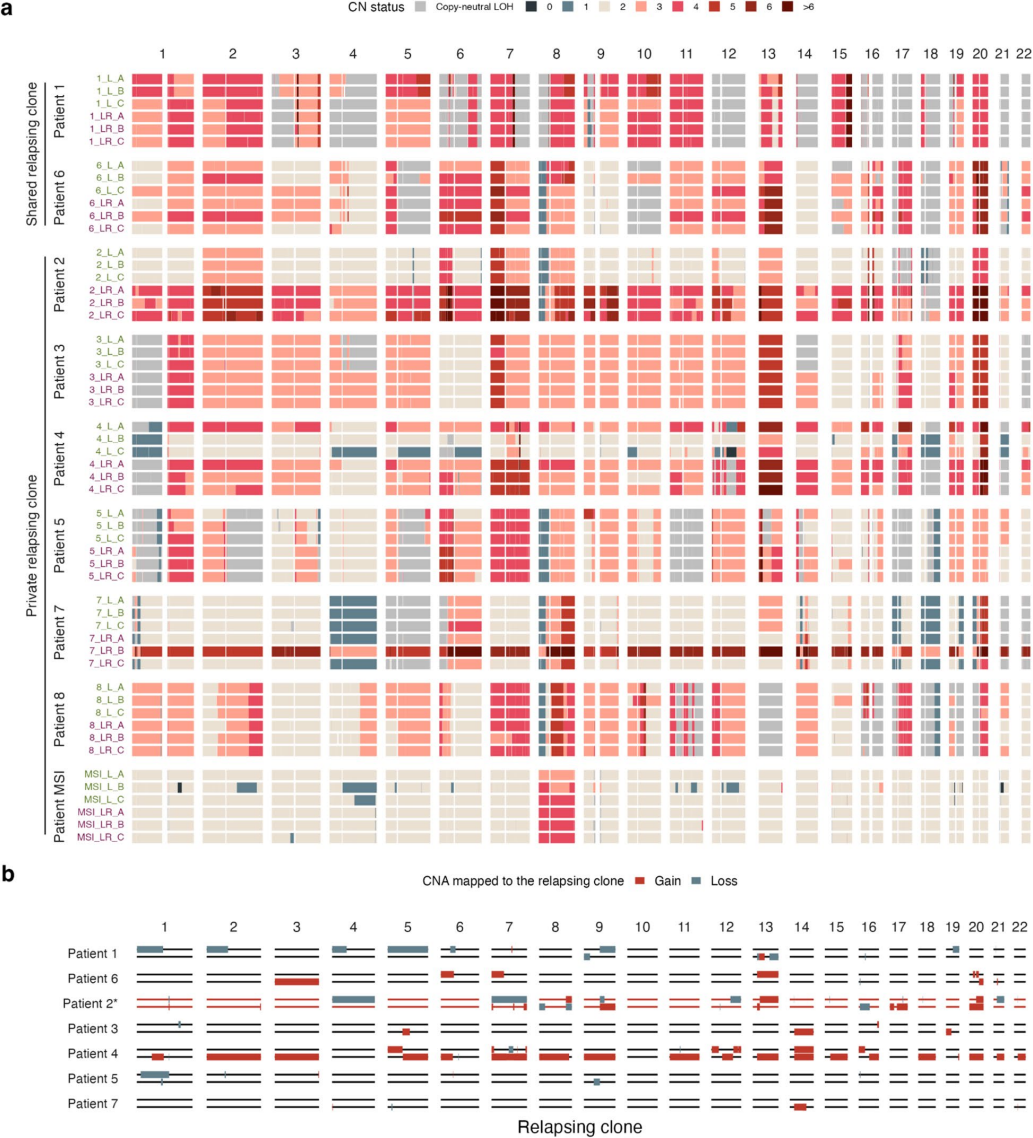
Somatic copy number alterations (SCNAs).** a** Overview of the SCNAs detected in each liver sample. Metastasis samples are labeled in green and relapse samples are labeled in purple. **b** SCNAs mapped to the relapsing clone of each patient according to MEDICC2 copy-number-based phylogenetic reconstruction. Horizontal lines represent the two alleles of the relapsing clone and solid boxes show the CNAs mapped to each of them. The asterisk and red lines in *Patient 2* indicate that the relapsing clone suffered a WGD, and the boxes represent only the additional CNAs. *Patient 8* and *Patient MSI* were excluded because the samples with the relapsing clone did not form a monophyletic clade in the MEDICC2 copy number tree (see Additional file 1: Fig. S6b)

All MSS tumors displayed marked aneuploidy, with copy number gains being particularly pervasive across all patients (Fig. 4a). Many of these SCNAs were already present in the initial metastases. Although relapse samples had a slightly higher average ploidy (3.27) compared to the corresponding metastases (2.75), this difference was not statistically significant (Wilcoxon paired test, *p* = 0.11) and could partly reflect technical biases. For instance, in *Patient 4*, the lower tumor cell content of the metastasis sample may have reduced SCNA detection sensitivity, whereas in *Patient 7*, the hexaploid relapse sample 7_LR_B might result from the non-identifiability between purity and ploidy (Additional file 1: Fig. S6a). Nonetheless, some changes between metastasis and relapse appear to be biological. For example, relapse samples from *Patient 2* exhibited evidence of a whole-genome duplication (WGD).

To further investigate copy number changes arising during relapse, we reconstructed copy-number-based phylogenies using MEDICC2. For most patients, the samples grouping was compatible with the relapsing pattern observed with the mutation-based clonal deconvolution, since the samples containing the relapsing clone formed a monophyletic clade (Additional file 1: Fig. S6b). We assumed that the branch leading to that clade contained the SCNA events of the relapsing clone. These events included the WGD in the relapsing clone of *Patient 2*, which also shows additional gains and losses (Fig. 4b). In addition, we found several losses in the relapsing clones of *Patient 1* and *Patient 5*, as well as multiple gains for *Patient 3*, *Patient 4*, *Patient 6*, and *Patient 7*. Notably, four patients exhibited copy number gains on chromosome 13 spanning the driver genes *BRCA2* and *NBEA* (Additional file 1: Fig. S6c).

In order to investigate whether other types of genomic alterations appeared exclusively after the relapse, we identified gene fusions using the transcriptome data. No candidate fusion was observed in more than one patient. Looking at each patient separately, we detected six gene fusion candidates in relapse samples, but none in the metastasis samples. Two of these candidates involved pseudo genes or open reading frames, and among the four remaining candidates, none were described in the fusion gene annotation update aided by deep learning FusionGDB2 [80] (Additional file 5: Table S4).

### Changes to neoantigen repertoire follow the relapsing clone pattern

**Fig. 5:**
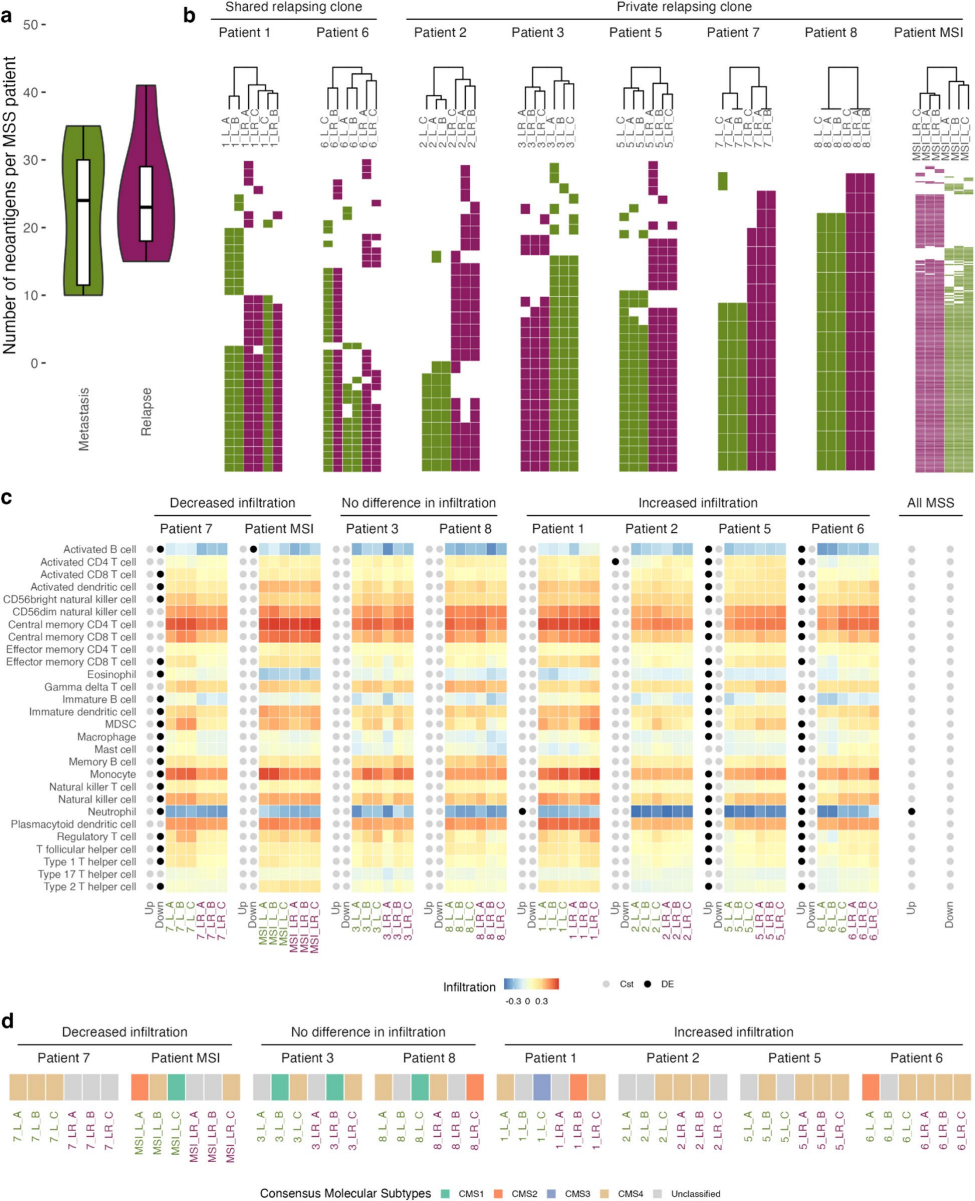
Neoantigens and transcriptomic profiles of tumor-infiltrating immune cells before and after relapse. **a** Number of neoantigens predicted by pVACseq in the metastasis and relapse samples of the MSS patients. Note: *Patient 4* is not included in any RNA analyses due to low tumor-cell content in the samples. **b** Neoantigens predicted in each sample. Samples are clustered based on the neoantigens' presence-absence profiles. **c** Patient-specific immune cell infiltration. Patients are grouped starting with reduced immune cell presence after relapse (decreased infiltration), no change after relapse (no difference in infiltration), and higher infiltration after relapse (increased infiltration). Immune cell profiles found differentially expressed after relapse are indicated in dark blue. **d** Consensus Molecular Subtypes (CMS) for each sample, patients grouped as in **c**

Our analysis unveiled minimal differences in the number of neoantigens between the metastasis and relapse samples across the entire cohort, with an average number of neoantigens of 21.7 before and 24.7 after relapse (Fig. 5a and Additional file 6: Table S5). The pattern of shared relapsing clones from *Patient 1* and *Patient 6* (Fig. 2) is repeated in the neoantigen profiles, whereby the samples do not cluster according to metastasis and relapse (Fig. 5b). Following relapse, the samples from *Patient MSI* demonstrated a surge in neoantigen count, nearly completely absent in the matched metastasis. Importantly, each patient had different tumor neoantigens, with no overlap across the cohort.

### Temporal heterogeneity in immune cell infiltration and CMS classification post-relapse is discordant and patient-specific

To explore whether the clonal evolution patterns extended to other measurable tumor features within our cohort, we looked at transcriptional profiles from the tumor samples. We examined alterations in the immune landscape after surgery and chemotherapy by assessing infiltrating immune cells in CRLM samples before and after relapse, using gene expression profiles. Samples from *Patient 4* were excluded from these analyses due to the low tumor cell content in the metastasis samples. We observed multiple infiltrating immune cells, including abundant monocytes and central memory T cells, while neutrophils and activated B cells remained low (Fig. 5c). When comparing the immune landscape of all samples before and after relapse across the cohort, only the neutrophil gene-set was differentially expressed in the model comprising all donors (p_adj_ = 0.028 after multiple testing correction, Additional file 7: Table S6). Our analysis revealed that immune cell infiltration profiles did not consistently align with the clonal evolution patterns.

A patient-by-patient comparison revealed significant changes in the immune cell subsets infiltrating the tumor. Patient samples, when grouped according to immune infiltration, ranged from significantly reduced after relapse, signifying a switch to colder tumor profiles, to significantly increased following relapse, signifying a switch to a hotter tumor environment (Fig. 5c). The *Patient 7* relapse samples displayed a significant reduction in subsets of infiltrating immune cells, while *Patient 3* and *Patient 8* samples remained relatively stable with no changes post-relapse. Activated B cells were reduced after relapse in *Patient MSI*, whereas samples from *Patient 1* and *Patient 2* exhibited an increase in neutrophils and CD4 T cells, respectively. Interestingly, we observed a significant increase in immune cell infiltration of relapse tumor samples from *Patient 5* and *Patient 6*.

Given the well-established CRC characterization into consensus molecular subtypes (CMS), we examined whether CRLM samples undergo changes in CMS following treatment and relapse. Using CMScaller [72], we found notable heterogeneity among samples derived from the same tumor. All the metastases contained at least one sample with the CMS4 profile (Fig. 5d). Samples from *Patient 7*, which displayed a large reduction in immune infiltration post-relapse, changed from a CMS4 profile across all metastasis samples to an unclassified profile after relapse. Samples from *Patient 3* and *Patient 8* remained relatively stable in CMS profiles before and after relapse, while samples from *Patient 2* with a slight increase in immune infiltration changed slightly toward a more CMS4 profile. Interestingly, the samples from *Patient 5* and *Patient 6* with increased infiltration after relapse changed their heterogeneous CMS profile completely to CMS4 in all relapse samples.

## Discussion

Recurrence after CRLM resection is common and often fatal, yet the origins of this relapse remain poorly understood. Here, we analyzed the genetic and immune microenvironmental features of liver metastasis relapse in nine patients with CRC. In these patients, relapse was seeded by a single clone in all cases, consistent with our previous single-cell study of CRLM and relapse[Citation error]. We identified different patterns of relapse: (i) in seven patients, the relapsing clone was undetectable in the pre-resection metastasis samples ("private relapsing clone"), while (ii) in two patients, the relapsing clone was already present in pre-resection metastases ("shared relapsing clone").

Private relapsing clones may have been seeded from extrahepatic sites, including residual tumors at the primary location or unsampled distant metastases [45, 81]. Alternatively, these relapses may have evolved from non-resected, residual tumor cells that continued accumulating mutations. In *Patients 2, 5,* and *MSI*, we found an ancestral clone shared between metastasis and relapse clones that was not detected in the primary lesion. This points towards a sequential dissemination trajectory with the relapsing clone originating in the initial liver metastasis. However, given that this analysis includes only a single primary tumor sample, we cannot rule out the possibility that the relapsing clone was present in the primary tumor. For *Patient 7*, we detected two different lineages in the metastasis and the relapse, a pattern compatible with independent branch-off seeding events.

The two patients with shared relapsing clones (*Patient 1* and *Patient 6*) display genetic patterns compatible with a sequential dissemination model. In this case, a plausible explanation is that these clones were not completely eradicated during surgery. Both patients had undergone 5FU treatment before metastasis resection, creating a selective pressure for resistant clones. Supporting this idea, all samples from *Patient 1* harbored a clonal *SMAD4* p.R361C mutation, recently linked to 5FU resistance in CRLM [37]. This suggests that the entire metastasis was already resistant, potentially due to the expansion of a resistant clone during the neoadjuvant treatment. *Patient 6* presented a temporary response to treatment prior to the resection of the metastasis, indicating a decrease in the tumor cell population. Both scenarios are compatible with 5FU treatment creating an evolutionary bottleneck, increasing the frequency of the mutations caused by 5FU and making the signature detectable.

Several studies have investigated primary and metastatic patterns of CRC dissemination. Chen et al. [45] reported sequential and branch-off patterns at initial stages of CRC progression, but they did not investigate relapsed liver metastases after surgery and treatment as we did in this study. Naxerova et al.[82] found that lymph node and distant metastases often arose from independent subclones within the primary tumor. Their study included a single case of relapsed liver metastasis in which the metastatic subclone gave rise to several liver metastases and a lymph node metastasis, consistent with the shared relapsing clone pattern observed in our data. Nikbakht et al. [35] described one patient with liver relapse and suggested that the two liver samples had similar mutational profiles. In our study, we confirmed that both the sequential and the branch-off patterns can occur in relapsed liver metastasis.

Using a multi-region sampling strategy combined with clonal deconvolution, we were able to reconstruct tumor evolutionary trajectories with greater resolution than previous studies. Earlier approaches often focused on comparing samples rather than tracing the evolution of clonal lineages. Standard studies that rely on unmatched pre- versus post-relapse comparisons [29, 36] can overlook shared relapsing clones, as these clones are only detectable when matched samples from the same patient are used. Similarly, studies that track genomic evolution using only multiregional sample trees [83] may miss crucial subclonal patterns responsible for relapse. With our approach, we saw that the relapsing clone of *Patient 6* diverged already in the primary tumor, was detected in one of the first liver metastasis samples, and later gave rise to the relapse. An independent clone stemming from the primary tumor was detected in the remaining two liver metastasis samples, suggesting at least two metastatic seeding events.

In relapsing clones, we identified alterations in COREAD driver genes linked to 5FU resistance, including *ARID1A *[84, 85] (*Patient 2*), *FAT4 *[86] (*Patient 3*), and *NCOR2 *[87] (*Patient MSI*). This suggests that undetected resistant clones may have repopulated the liver following selective pressure from chemotherapy. Notably, only the samples with a shared relapsing clone (*Patient 1* and *Patient 6*) exhibited a mutational signature associated with 5FU [88]. This signature was absent in a similar study comparing pre- and post-5FU treatment samples [29], but we previously detected it in the relapsing clone of our single-cell study[Citation error]. These findings could mean that 5FU itself may have contributed to drug resistance by inducing resistance mutations, a phenomenon described in other cancer contexts with chemotherapy [89, 90]. Additionally, we found an amplification of chromosome 13 involving genes *BRCA2* and *NBEA* in the relapsing clone of four different patients, and a WGD in the relapsing clone of *Patient 2.* However, the majority of copy number alterations were present both before and after relapse, in agreement with prior observations suggesting that chromosomal instability occurs early and remains clonal in CRC [91].

Inevitably, our small cohort of nine patients may restrict the generalizability of our results. Including additional patients and tumor samples from different anatomical sites could provide further insights into dissemination trajectories. We were able to incorporate primary tumor samples from five patients; however, these samples were single-region and derived from FFPE archival blocks. While inclusion of the primary tumors provided more information about the evolutionary trajectory, the potential FFPE-related sequencing artifacts prevented the inclusion of these samples in several analyses. A more comprehensive sampling of the primary tumor, including multi-regional and fresh-frozen or high-quality preserved material, would reduce uncertainty in the absence of mutations due to sampling bias [92]. A larger cohort would be necessary to investigate other patterns of relapse, and how they relate to different treatment strategies.

As immunotherapies have taken on an important role in cancer treatment, many studies have attempted to predict the response of otherwise ‘cold’ colorectal cancer to checkpoint inhibitors [17, 26]. Previous work has shown that increased immune cell infiltration is associated with a favorable response to checkpoint inhibitors [20, 93]. In our cohort, *Patient 5* and *Patient 6*, whose samples exhibited a notable increase in leukocyte abundance after relapse, may be considered candidates for this treatment, particularly since their tumors transitioned to a more CMS4 profile after relapse, a change that has been predicted to reduce response to cytotoxic treatment [24, 37, 94]. To further assess tumor immunogenicity a more detailed analysis would be recommended [69]. However, due to limited tumor cell purity, this was not feasible in our study. Moreover, although previous studies have linked immune infiltration to mutational burden and neoantigen load [95], we did not observe this relationship in our cohort. A more thorough study of individual patient samples should be considered when developing personalized therapy protocols, which may include checkpoint inhibitors in the later stages of MSS colorectal cancer.

## Conclusions

In this study, we investigated the evolutionary trajectories of colorectal cancer liver metastases and their relapse following surgery alone or surgery combined with systemic therapy in nine patients. We identified two distinct patterns of clonal evolution: in one group of patients, liver metastases already contained a clone that was subsequently detected in all relapse samples, whereas in the other group no evidence of relapse-associated clones was observed at the metastatic stage. Relapse-associated clones detected in the metastasis exhibited a distinct DNA mutational signature attributable to 5-fluorouracil exposure. In contrast, transcriptional analyses of immune infiltration revealed no consistent evolutionary pattern, with patient samples showing heterogeneous changes ranging from reduced or unchanged immune infiltration to increased immune cell presence at relapse. Together, these results demonstrate that genomic evolution of colorectal cancer metastases may be shaped by therapeutic exposure, while immune remodeling at relapse remains highly patient-specific.

## Supplementary Information

Additional file 1: Supplementary_Figures.

Additional file 2: Table S1: Cohort description.

Additional file 3: Table S2: DNA mutations and SCNAs.

Additional file 4: Table S3: Mutation clusters.

Additional file 5: Table S4: Fusion gene analysis.

Additional file 6: Table S5: Neoantigens.

Additional file 7: Table S6: Immunome.

## Data Availability

The data generated in this study are available via controlled access in the German Human Genome-Phenome Archive (GHGA, data.ghga.de) under GHGA Accession GHGAS92185214452127 [96] (https://paperpile.com/c/ewGI7C/3iFl). Further details, including the data access policy for the study, can be found there. [https://data.ghga.de/study/GHGAS92185214452127] (https://data.ghga.de/study/GHGAS92185214452127). The code is available from GitHub [97] (https://github.com/lautomas/LiverRelapse) and Zenodo [98] (10.5281/zenodo.18461364).

## Abbreviations

5-FU: 5-Fluorouracil
CMS: Consensus molecular subtypes
COREAD: Colorectal adenocarcinoma compendium driver genes
CRC: Colorectal cancer
CRLM: Colorectal liver metastasis
FFPE: Formalin-fixed paraffin-embedded
GATK: Genome Analysis Toolkit
Indels: Insertions and deletions
MSI: Microsatellite instable
MSS: Microsatellite stable
SBS: Single-base substitution
SCNA: Somatic copy number alteration
SNV: Single-nucleotide variant
VAF: Variant allele frequency
WES: Whole-exome sequencing
WGD: Whole-genome duplication
