# Supplementary material for: Cancer evolution and multi-omic profile of relapsed colorectal liver metastases after treatment
Source: Genome Med. 2026 Feb 24;18:28. doi: 10.1186/s13073-026-01614-0 (PMC12980905; doi:10.1186/s13073-026-01614-0)
Supplement: Supplementary file 1 — Additional file 1: Supplementary_Figures. [file 13073_2026_1614_MOESM1_ESM.pdf]

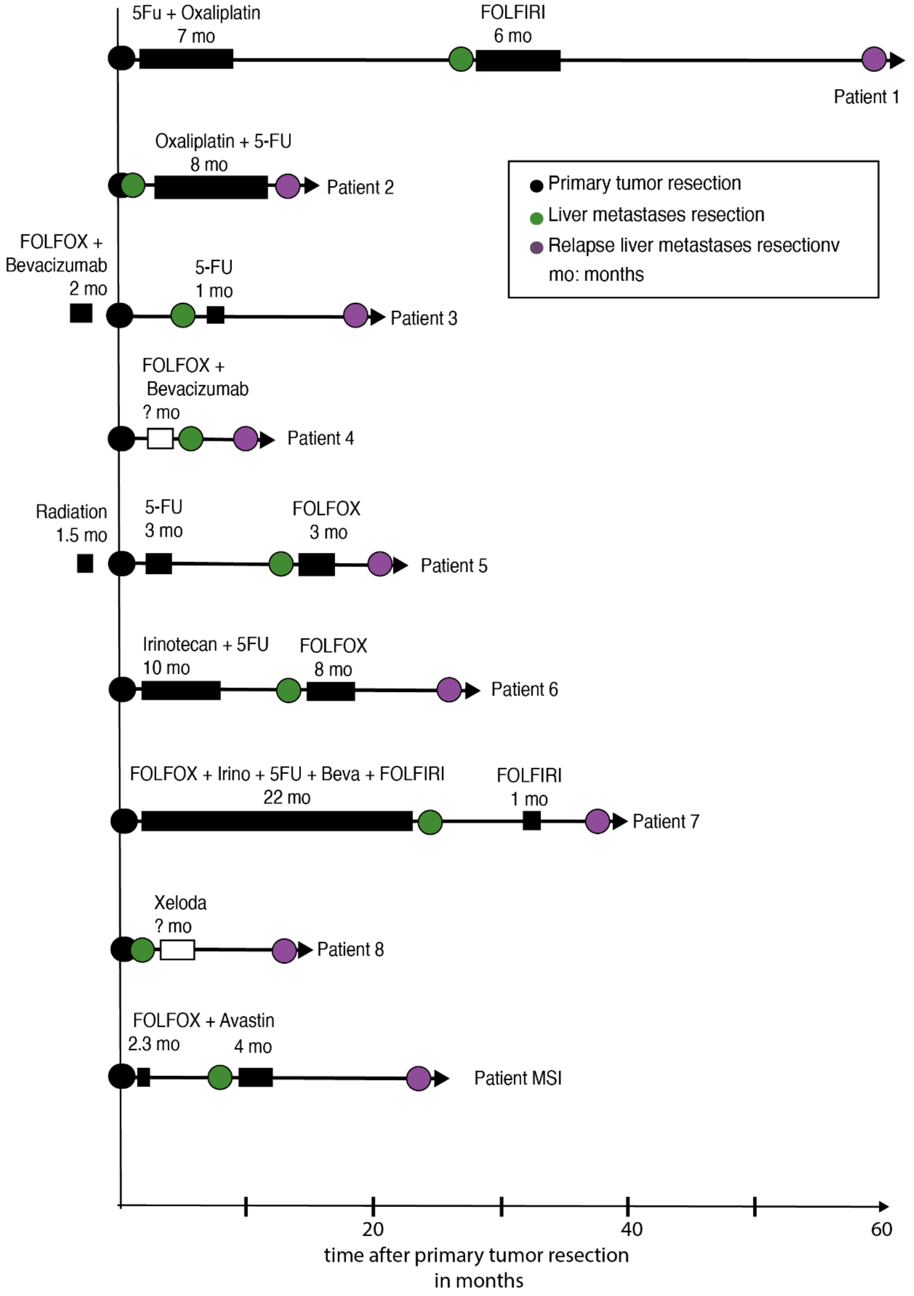

Figure S1. Detailed treatment history of the patients. Treatment represented by black boxes, circles represent the resection dates. Time timeline begins with primary tumor resection.

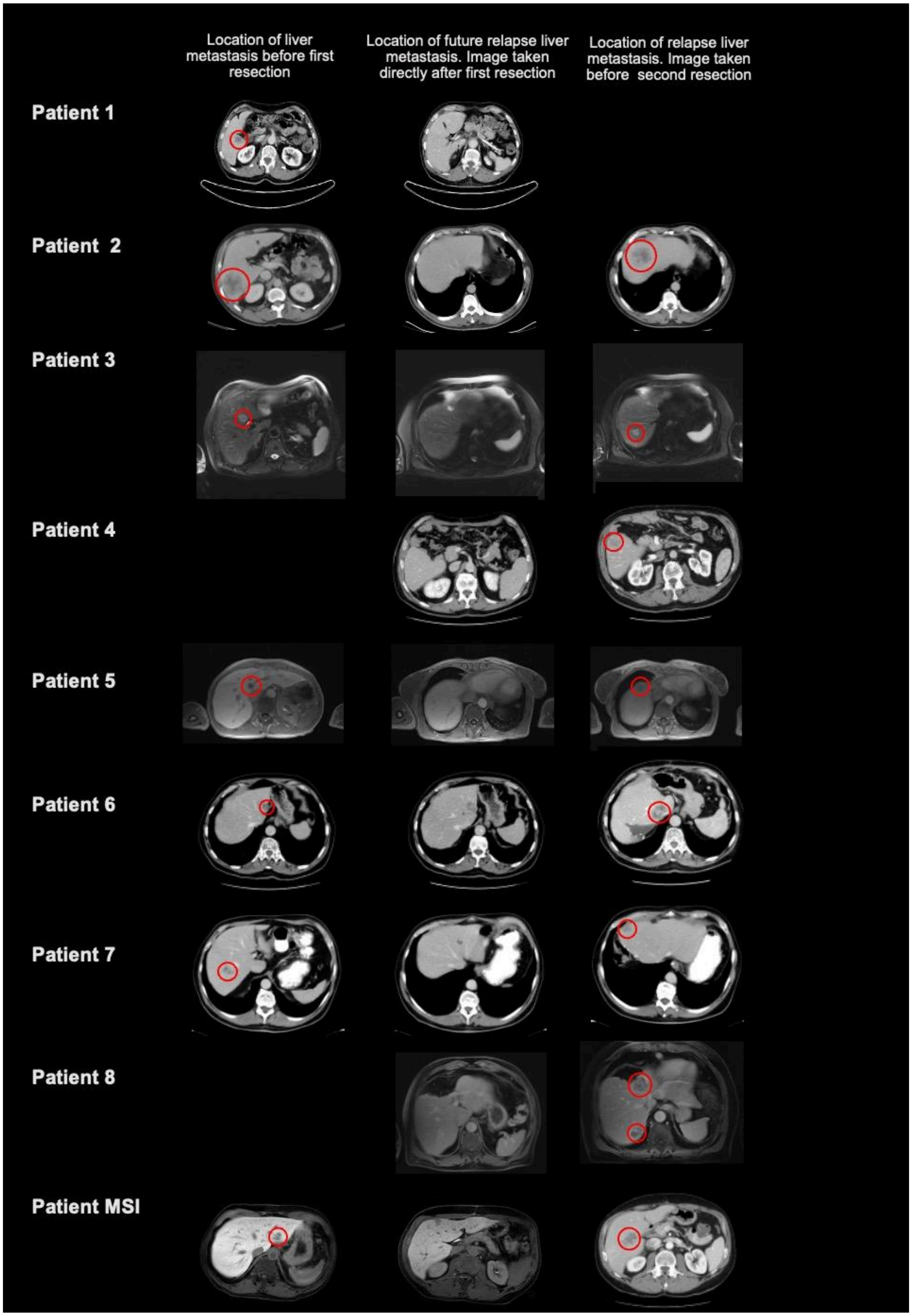

Figure S2. CT scans of liver segments of tumor locations before metastases resection, directly after metastases resection and before relapse liver metastases resection. Red circles mark tumor areas resected and included in our study. Missing figures were not available.

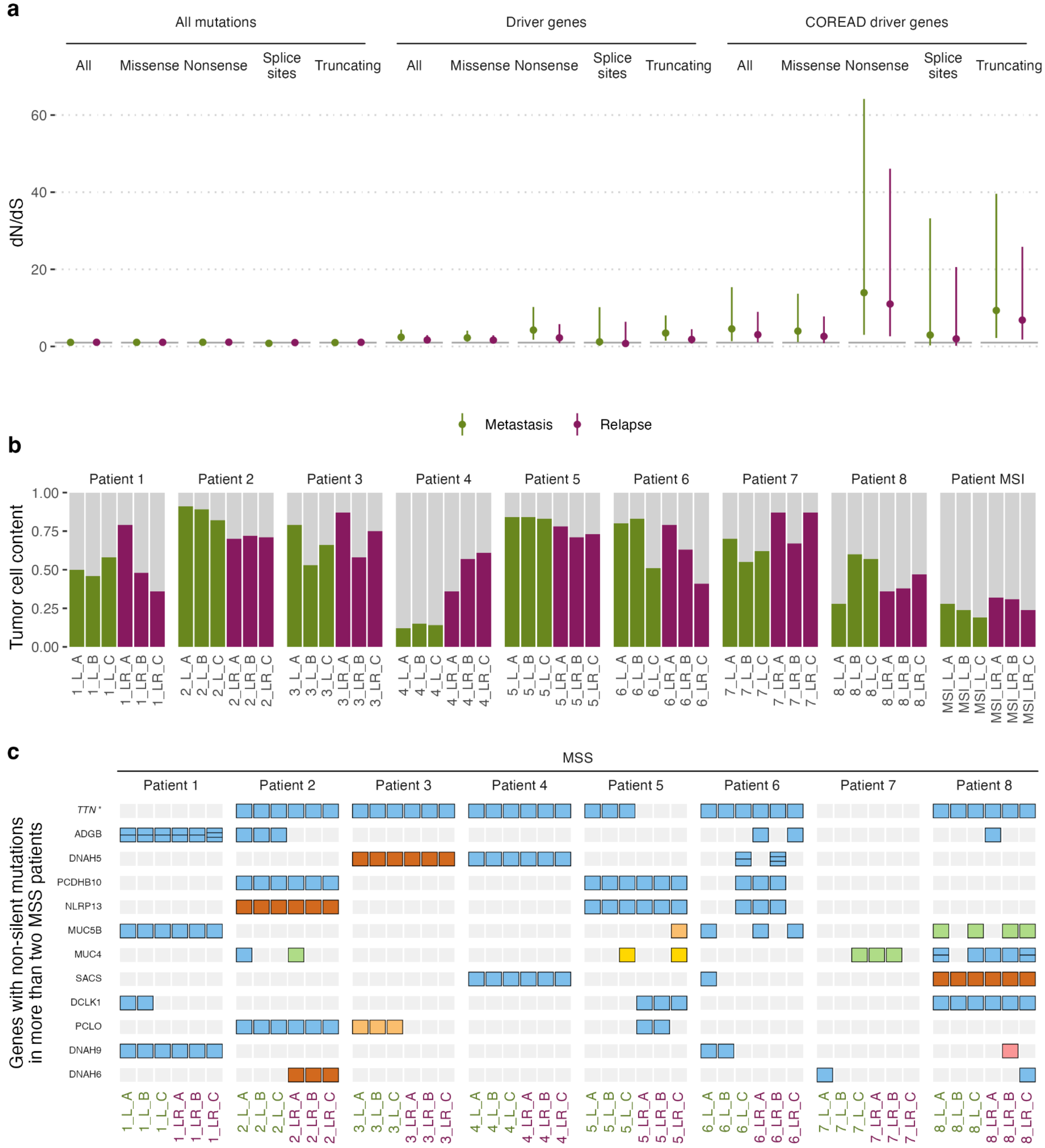

Figure S3. a. dN/dS values for the mutations found in the metastasis (green) and the relapse (purple) samples of the MSS patients. **b.** Tumor cell content of each sample. **c.** Genes with non-silent mutations in more than two MSS patients from the cohort, excluding the COREAD driver genes.

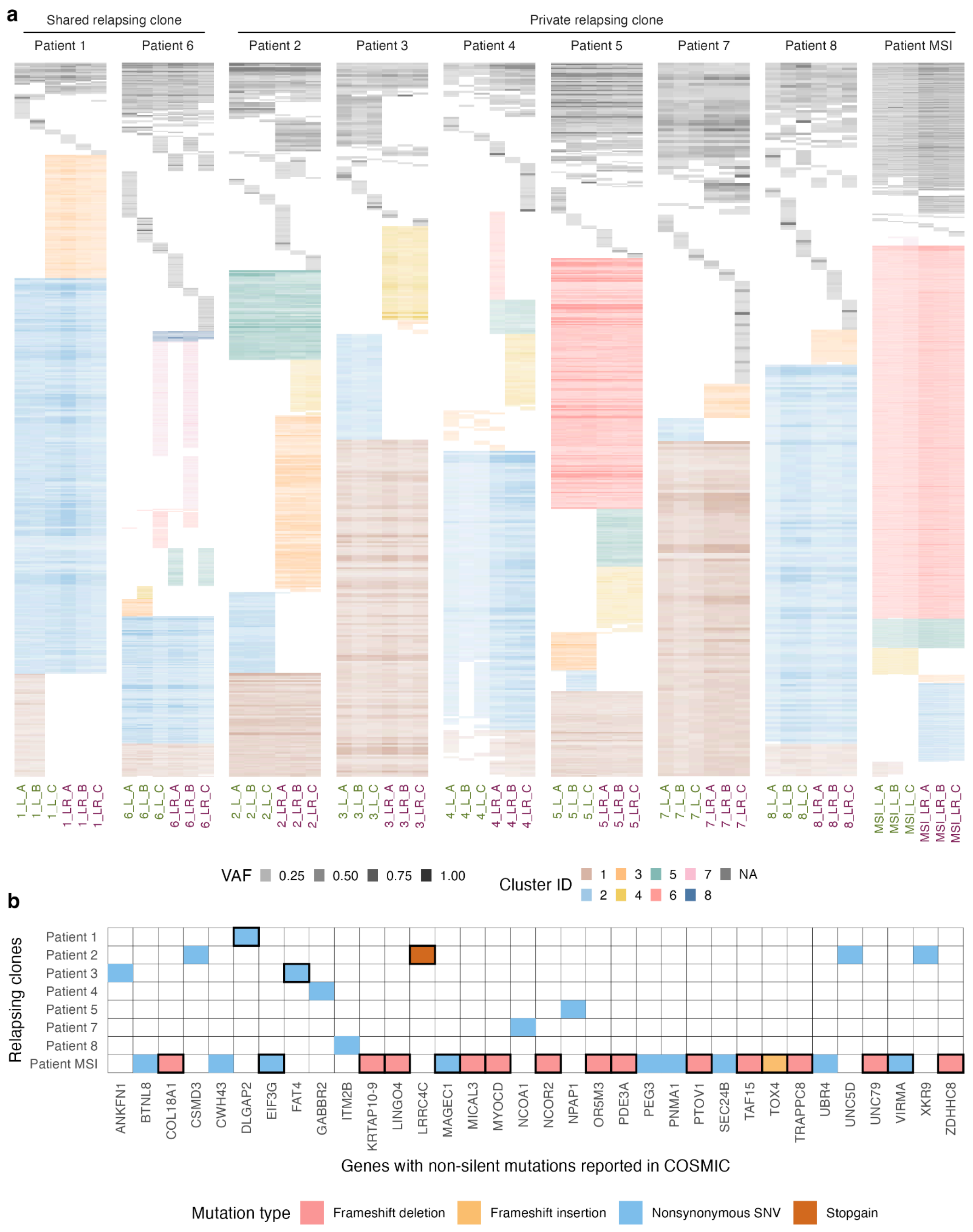

Figure S4. a. An overview of all mutations in metastasis and relapse samples color-coded by cluster ID. Mutations not assigned to any cluster are depicted in gray. **b.** Non-silent mutations harbored by the relapsing clones that have been reported in COSMIC. Mutations reported in COSMIC in "large intestine" cases are outlined in black.

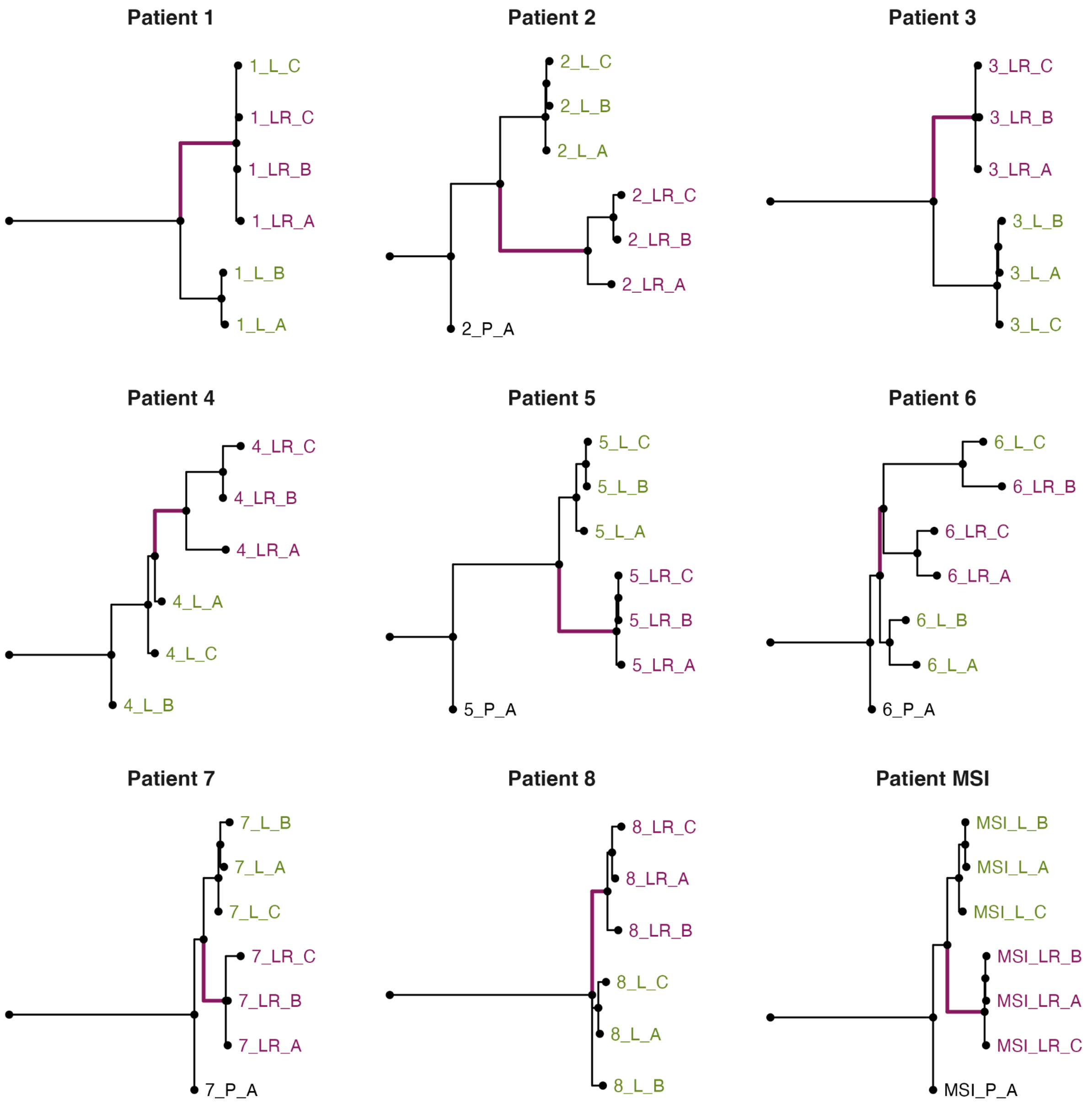

Figure S5. Mutation-based sample tree reconstruction with Treeomics. This analysis includes FFPE primary tumor samples when available, but only mutations detected in at least one tumor samples were retained.

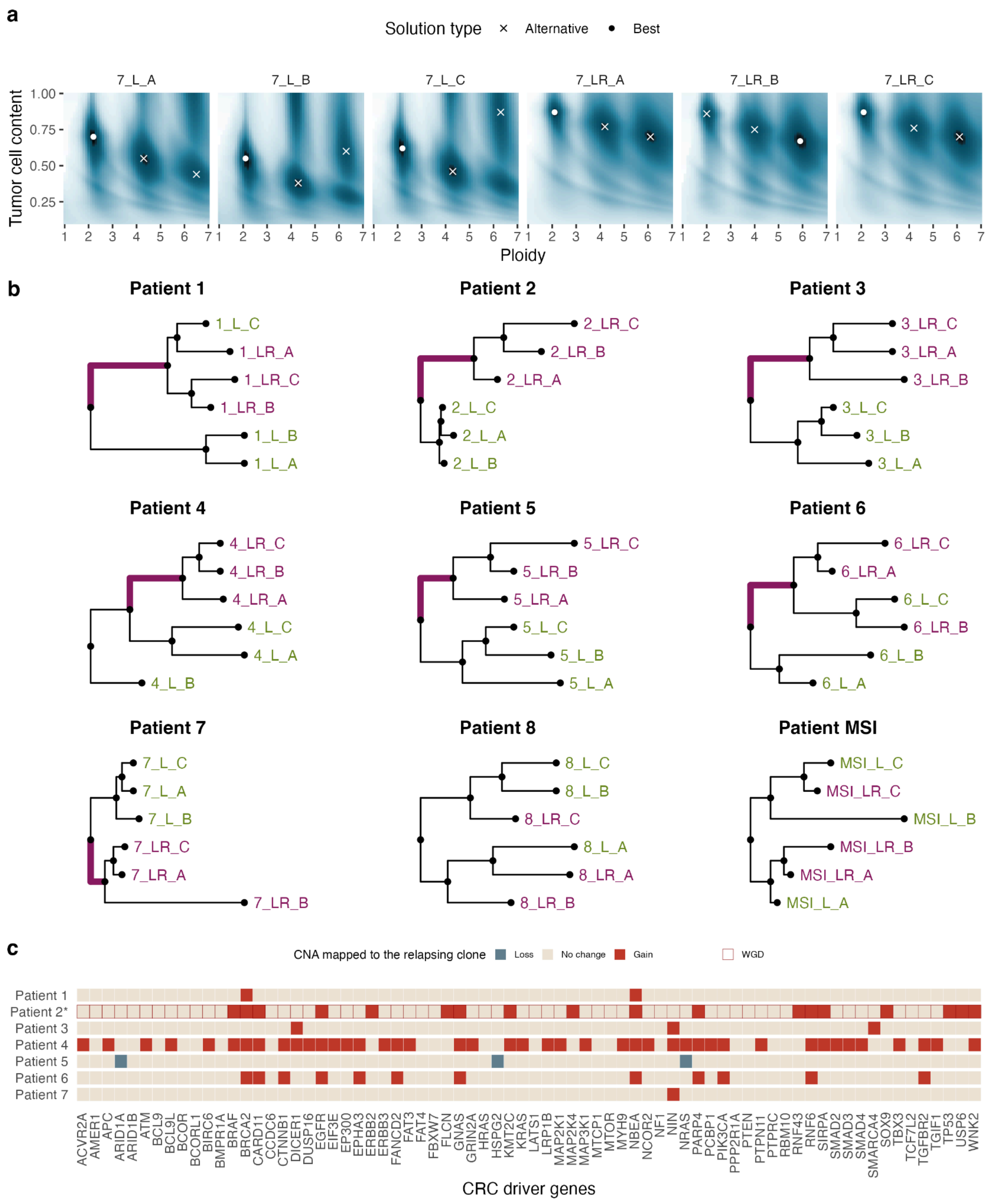

Figure S6. SCNAs and copy-number-based phylogenetic reconstruction. a. Likelihood of each tumor cell content and ploidy combinations as reported by Sequenza for *Patient 7*. The highest-likelihood solution is indicated with a dot, while other possible solutions are marked with a cross. The best solution for sample 7_LR_B implies a ploidy of six, but there is a high-likelihood alternative solution with ploidy two, which would match the ploidy in the other samples from the patient. **b.** Copy-number-based sample tree reconstruction with MEDICC2. FFPE tumor samples were excluded from this analysis due to the low quality of their copy number profiles. Branches compatible with the relapsing clone detected in the mutation-based clonal deconvolution are highlighted in purple. **c.** CNAs mapped to the relapsing clone that affect COREAD driver genes.
